# Pericarditis caused by *Mycobacterium africanum*: case report

**DOI:** 10.1186/s12879-022-07540-x

**Published:** 2022-07-18

**Authors:** Paula Mascarell, Alba de la Rica, Sergio Padilla, Montserrat Ruiz-García, José López-Escudero, Javier García-Abellán, Ángela Botella, Mar Masiá, Félix Gutiérrez

**Affiliations:** 1 Infectious Diseases Unit, Hospital General Universitario de Elche, Alicante, Spain; 2 Microbiology Service, Hospital General Universitario de Elche, Alicante, Spain

**Keywords:** *Mycobacterium africanum*, *Mycobacterium tuberculosis complex*, Tuberculous pericarditis, Interferon-gamma release assays, Case report

## Abstract

**Background:**

*Mycobacterium africanum* is a member of the *Mycobacterium tuberculosis complex* (MTBC) and is endemic in West Africa, where it causes up to half of all cases of pulmonary tuberculosis. Here, we report the first isolation of *Mycobacterium africanum* from the pericardial effusion culture of a patient with tuberculous pericarditis.

**Case presentation:**

A 31-year-old man, native from Senegal, came to the emergency room with massive pericardial effusion and cardiac tamponade requiring pericardiocentesis. *M. africanum* subtype II was identified in the pericardial fluid. The patient completed 10 months of standard treatment, with a favorable outcome.

**Conclusions:**

We report the first case of tuberculous pericarditis caused by *Mycobacterium africanum*, which provide evidence that this microorganism can cause pericardial disease and must be considered in patients from endemic areas presenting with pericardial effusion.

## Background

*Mycobacterium africanum (M. africanum)* is a member of the *Mycobacterium tuberculosis complex* (MTBC) and a primary cause of human tuberculosis disease globally [1]. The MTBC consists of seven phylogenetically distinct lineages (L1–L7) and the phylogenetic study of *M. africanum* classifies it into two groups of different biochemical characteristics and geographical origin: *M. africanum subtype I* (L5) and *M. africanum subtype II* (L6) [2, 3].

*M. africanum* is endemic in West Africa, where it causes up to half of all cases of pulmonary tuberculosis [4]. In developed countries, cases are mostly restricted to migrants from endemic areas [5]. Although the pulmonary form has predominantly been described, some cases of extrapulmonary presentations (pleural,cutaneous, bone, cerebral, prostatitis or epididymitis) have been published [6–11]. To the best of our knowledge, no cases of pericardial involvement have ever been reported.

We describe the first case of tuberculous pericarditis caused by *M. africanum.* The patient presented with massive pericardial effusion and cardiac tamponade requiring pericardiocentesis and *M. africanum* subtype II was identified in the pericardial fluid.

### Case presentation

A 31-year-old man, native from Senegal and living in Spain for one year, came to the emergency room reporting non-productive cough, asthenia, evening fever and profuse sweating of one month duration, with progressive dyspnea limiting ordinary activity from the day before admission.

Patient’s condition met the Beck’s triad including arterial hypotension (a blood pressure of 85/65 mmHg), muffled heart sounds and mild jugular engorgement.

**Fig. 1:**
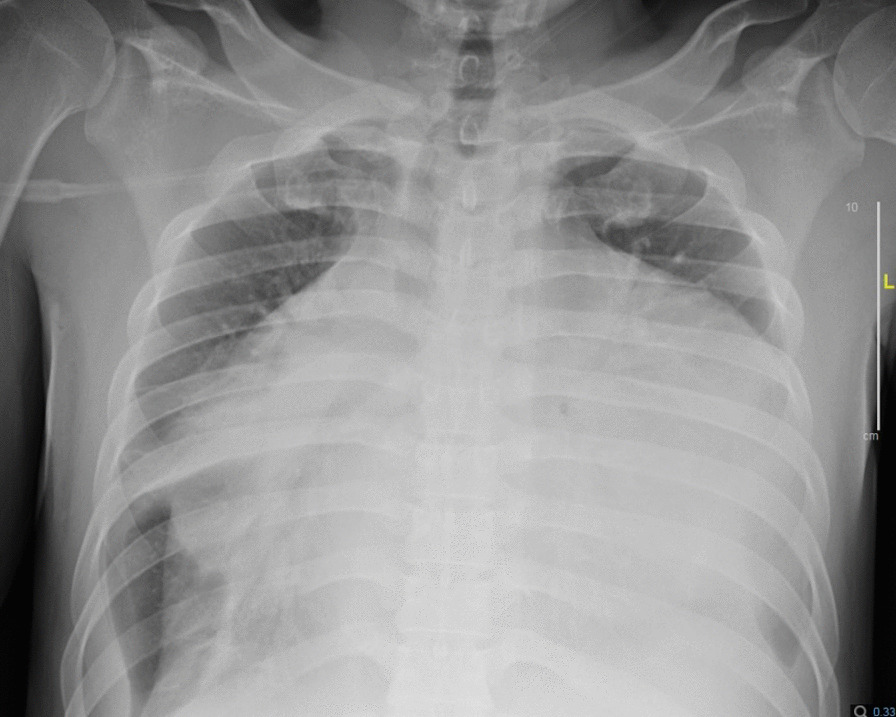
Chest x-ray showing a significant increase in the cardiac silhouette with bilateral pleural effusion without pulmonary lesions

**Fig. 2:**
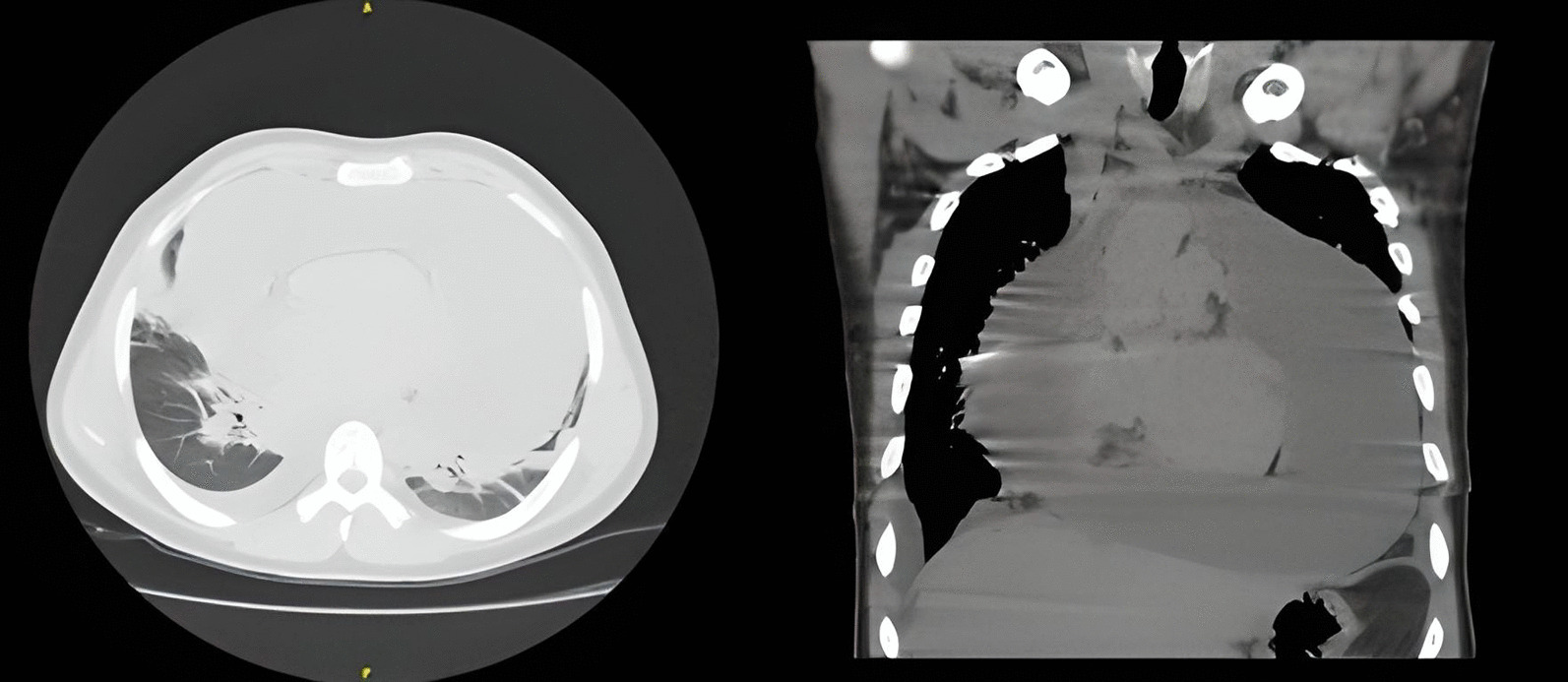
Chest computed tomography showing a massive pericardial effusion

Chest X-ray (Fig. 1) and computed tomography (Fig. 2) revealed a significant increase in the cardiac silhouette with bilateral pleural effusion and without pulmonary lesions. The electrocardiogram showed a heart rate of 100 beats per minute, with low voltage, a right bundle branch block and electrical alternans.

**Fig. 3:**
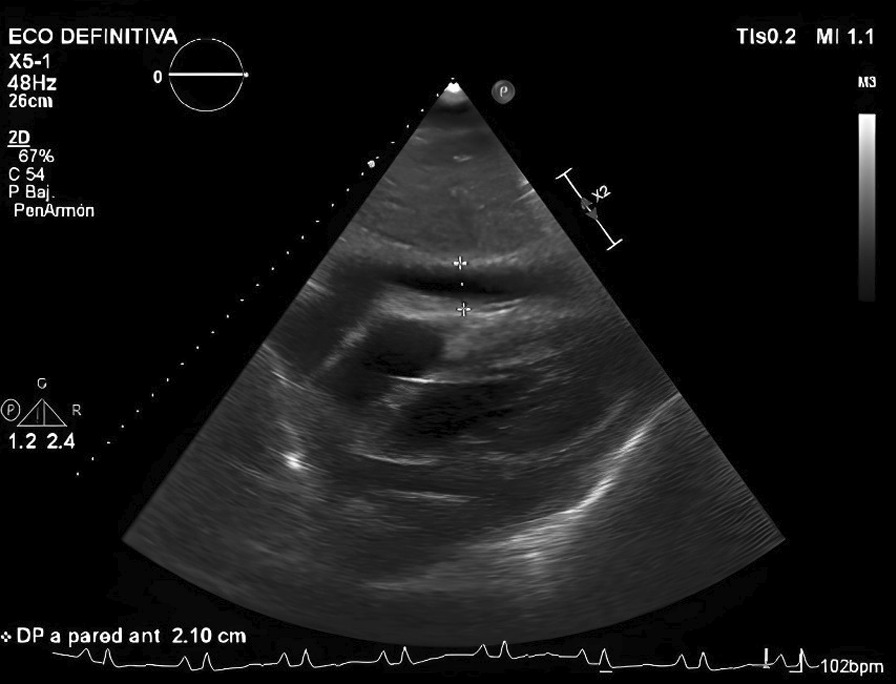
Transthoracic echocardiography showing a massive pericardial effusion

Transthoracic echocardiography described severe pericardial effusion with echocardiographic signs of hemodynamic compromise, right ventricular diastolic collapse, and right atrium systolic collapse, as well as a dilated inferior vena cava and global hypokinesia that conditioned a severely depressed left ventricular ejection fraction (30%) (Fig. 3).

To investigate the etiology of pericardial effusion and cardiac tamponade, an ultrasound-guided percutaneous puncture pericardiocentesis and pericardial biopsy with a pericardioscope through the subxiphoid route were performed. Pericardial effusion had serohematic appearance and the cytochemical and cytological study showed increased cells, predominantly lymphocytes and macrophages, with elevated adenosine deaminase levels (120,2 U/L). Conventional bacteriologic cultures of pericardial effusion were negative. Acid-alcohol-fast bacilli were not observed in the Ziehl-Neelsen stain. The pericardial sample was processed for culture in liquid medium (Bactec MGIT 960) and in enriched solid medium (Coletsos and Löwenstein-Jensen). Since only one sample was available for all microbiological processes, direct PCR and liquid culture were performed from the same sample. The direct MTBC PCR (FluoroTypeMTB-Hain) from the pericardial effusion was negative. Liquid medium culture was positive for mycobacteria after seven weeks of incubation, and PCR performed at this moment from this liquid medium was also positive for MTBC. Rapid confirmation of presumptive positive liquid cultures of Mycobacterium tuberculosis, based on the detection of the MPT64 antigen, was negative from the liquid medium, but positive from direct colony.

The strain was sent to the Mycobacteria Laboratory of the National Center for Microbiology (Carlos III Health Institute), where it was identified as *M. africanum*, and it showed to be sensitive to isoniazid, rifampin, pyrazinamide, streptomycin, and ethambutol.

Genomic epidemiologic analysis was carried out (Tuberculosis Genomics Unit of the Biomedicine Institute of Valencia) using the whole genome sequencing technique for a subsequent genomic analysis of single nucleotide point mutations (SNP). The species and lineage identified were *M. africanum* subtype *II.* The analysis of genetic distances showed it was a single sample, not belonging to any transmission group of the Valencian Community included in the Tuberculosis Genomics Unit’s database. The strain was found at a distance greater than 300 SNPs from the closest strain. No specific SNPs were identified that confirmed resistance to the different first and second line antituberculosis drugs.

IFN-γ production was determined by *QuantiFERON-TB Gold Plus (QFT-Plus)* in the patient’s blood using ESAT-6 and CFP-10 antigens and was negative.

Pericardial biopsy revealed non-necrotizing chronic granulomatous pericarditis.

The HIV serology test was negative.

Antituberculous therapy including isoniazid, rifampin, ethambutol, and pyrazinamide was started, as well as adjuvant treatment with corticosteroids.

The patient was switched to isoniazid and rifampicin after an initial two-month regimen with four drugs and completed 10 months of therapy, with a favorable outcome. The patient underwent periodic transthoracic echocardiograms. One month after hospital admission, the pericardial effusion had improved, and a preserved left ventricular ejection fraction was observed.

### Discussion and conclusions

Pericarditis is a rare manifestation of tuberculosis that carries high morbidity and mortality despite proper diagnosis and treatment. Tuberculous etiology represents less than 4% of all cases of pericarditis in developed countries [12]. The pericardium is affected by contiguity from the peritracheal, peribronchial and mediastinal nodes, or by the hematogenous route from pulmonary tuberculosis.

Although pulmonary and extrapulmonary tuberculosis caused by *M. africanum* have been reported, this is the first ever-reported case of pericardial involvement, causing tuberculous pericarditis with cardiac tamponade. *M. africanum* infrequently causes tuberculosis infection in Spain. Tuberculosis disease caused by *M. africanum* is more frequently associated with elderly patients, severe malnutrition, HIV infection and with severe abnormal radiological findings in the X-ray [13]. *M. africanum* induces similar clinical manifestations to the rest of mycobacteria belonging to the MTBC, although it has lower virulence and lower rate of progression to active tuberculosis than *M. tuberculosis* [14]. *M. africanum* lineages show microaerobic growth and are usually associated with extrapulmonary disease, which suggests a preference for regions with low oxygen [15].

Crucial virulence mechanisms have been shown to be altered in *M. africanum* infection, including an attenuation of T-cell responses to the mycobacterial-secreted antigenic and virulence factor ESAT-6, that could impair the accuracy of interferon-gamma release assays (IGRA) [16]. This would explain the negative results of the *QuantiFERON-TB Gold Plus* test observed in our patient.

Interferon-gamma release assays have recently shown promising results in diagnosing active extrapulmonary tuberculosis. The T-SPOT.TB has shown a high sensitivity and specificity on tuberculosis pericardial effusion [17]. Therefore, these tests appear to be a promising rapid diagnostic method with high diagnostic accuracy for diagnosis of tuberculous pericarditis [18].

*M. africanum* grows more slowly than *M. tuberculosis*; it can take up to 10 weeks to yield growth in cultures [19].

Regarding treatment, *M. africanum* infections respond to regular tuberculosis treatment. Adjuvant steroid treatment has been associated with a decrease in the incidence of pericardial constriction and hospitalization in mycobacterial pericarditis [20].

In conclusion, *M. africanum* can cause pericardial disease and must be considered in patients from endemic areas presenting with pericardial effusion. The diagnosis may be challenging due to the longer growth time than *M. tuberculosis* and the fact that the disease is less likely to have a positive IGRA result. These difficulties become more relevant in areas where there is a greater distribution of *M. africanum* and sometimes with fewer available resources, as in the case of Africa.

## Data Availability

Not applicable.

## Abbreviations

MTBC: *Mycobacterium tuberculosis complex*
*M. africanum*: *Mycobacterium africanum*
PCR: Polymerase chain reaction
SNP: single nucleotide point mutations
IGRA: interferon-gamma release assays

## References

[1] Thorel MF (1980). Isolation of *Mycobacterium africanum* from monkeys. Tubercle.

[2] Gagneux S, DeRiemer K, Van T, Kato-Maeda M, de Jong BC (2006). Variable host-pathogen compatibility in *Mycobacterium tuberculosis*. Proc Natl Acad Sci U S A.

[3] Coscolla M, Gagneux S, Menardo F (2021). Phylogenomics of *Mycobacterium africanum* reveals a new lineage and a complex evolutionary history. Microb Genom.

[4] De Jong BC, Antonio M, Gagneux S (2010). *Mycobacterium africanum*-review of an important cause of human tuberculosis in West Africa. PLoS Negl Trop Dis.

[5] Isea-Peña MC, Brezmes-Valdivieso MF, Gonzalez-Velasco MC, Lezcano-Carrera MA, Lopez-Urrutia-Lorente L, Martin-Casabona N (2012). *Mycobacterium africanum*, an emerging disease in high-income countries?. Int J Tuberc Lung Dis.

[6] Aldea MJ, Lezcano MA, Esteban A, Bello S, Vila M (1990). Pleuropulmonary tuberculosis caused by *Mycobacterium africanum* in a White male. Enferm Infecc Microbiol Clin.

[7] Baril L, Caumes E, Truffot-Pernot C, Bricaire F, Grosset J (1995). Tuberculosis caused by *Mycobacterium africanum* associated with involvement of the upper and lower respiratory tract, skin, and mucosa. Clin Infect Dis.

[8] Petit JC, Lesage D (1980). Bone tuberculosis due to *Mycobacterium africanum*-2 cases. Nouvelle Presse Med.

[9] Bhanot N, Badem O, Mathew L, Haran M (2008). *Mycobacterium africanum* presenting as a brain mass. Infect Dis Clin Pract.

[10] Remacha Esteras MA, Parra Parra I, Blanco Mercade MD (2003). Disseminated tuberculosis due to *Mycobacterium africanum*. Arch Bronconeumol.

[11] Pérez-de Pedro I, Bermúdez P, Arter I, Soledad Jiménez M (2008). Orquiepididimitis por *Mycobacterium africanum*. Enferm Infecc Microbiol Clin.

[12] Sagrista-Sauleda J, Permanyer-Miralda G, Soler-Soler J (1988). Tuberculous pericarditis: ten-year experience with a prospective protocol for diagnosis and treatment. J Am Coll Cardiol.

[13] De Jong BC, Adetifa I, Walther B (2010). Differences between tuberculosis cases infected with *Mycobacterium africanum*, West African type 2, relative to Euro-American *Mycobacterium tuberculosis*: an update. FEMS Immunol Med Microbiol.

[14] De Jong BC, Hill PC, Aiken A, Awine T, Antonio M, Adetifa IM, Jackson-Sillah DJ, Fox A, Deriemer K, Gagneux S, Borgdorff MW, McAdam KP, Corrah T, Small PM, Adegbola RA (2008). Progression to active tuberculosis, but not transmission, varies by *Mycobacterium tuberculosis* lineage in The Gambia. J Infect Dis.

[15] Ofori-Anyinam B, Riley AJ, Jobarteh T (2020). Comparative genomics shows differences in the electron transport and carbon metabolic pathways of *Mycobacterium africanum* relative to *Mycobacterium tuberculosis* and suggests an adaptation to low oxygen tension. Tuberculosis (Edinb).

[16] Jong BC, Hill PC, Brookes RH (2006). Mycobacterium africanum elicits an attenuated T cell response to early secreted antigenic target, 6 kDa, in patients with tuberculosis and their household contacts. J Infect Dis.

[17] Cho OH, Park KH, Kim SM, Park SJ, Moon SM, Chong YP (2011). Diagnostic performance of T-SPOT. TB for extrapulmonary tuberculosis according to the site of infection. J Infect.

[18] Bian S, Zhang Y, Zhang L, Shi X, Liu X (2016). Diagnostic value of interferon-γ release assays on pericardial effusion for diagnosis of tuberculous pericarditis. PLoS ONE.

[19] Grosset J, Decroix G, Sors C (1971). Tuberculosis due to *Mycobacterium africanum* in African negroes in the Paris area. Rev Tuberc Pneumol (Paris).

[20] Mayosi BM, Ntsekhe M, Bosch J (2014). IMPI Trial Investigators. Prednisolone and *Mycobacterium indicus pranii* in tuberculous pericarditis. N Engl J Med.

